# Automated, Quantitative Capillary Western Blots to Analyze Host Cell Proteins in COVID-19 Vaccine Produced in Vero Cell Line

**DOI:** 10.3390/vaccines12121373

**Published:** 2024-12-05

**Authors:** Paul F. Gillespie, Yanjie Wang, Kuo Yin, Emily Groegler, Nicholas Cunningham, Alyssa Q. Stiving, Jessica Raffaele, Natalia Marusa, Christopher M. Tubbs, John W. Loughney, Michael A. Winters, Richard R. Rustandi

**Affiliations:** 1 Analytical Research & Development, Merck & Co., Inc., Rahway, NJ 07065, USA; yanjie.wang@merck.com (Y.W.); kuoyin@gmail.com (K.Y.); emily.groegler@merck.com (E.G.); nicholas.cunningham@merck.com (N.C.); jessica.raffaele@merck.com (J.R.); natalia.marusa@merck.com (N.M.); christopher.tubbs@merck.com (C.M.T.); john_loughney@merck.com (J.W.L.); richard_rustandi@merck.com (R.R.R.); 2 Process Research & Development, Merck & Co., Inc., West Point, PA 19486, USA; michael_winters@merck.com

**Keywords:** host cell protein (HCP), capillary western, Simple Western^TM^, V590, COVID-19

## Abstract

Background/Objectives: Host cell protein (HCP) content is a major attribute for biological and vaccine products that must be extensively characterized prior to product licensure. Enzyme Linked Immunosorbent Assay (ELISA) and Mass Spectrometry (MS) are conventional methods for quantitative host cell protein analysis in biologic and vaccine products. Both techniques are usually very tedious, labor-intensive, and challenging to transfer to other laboratories. In addition, the ELISA methodology requires 2D SDS PAGE and 2D western blot antibody reagent validation to establish reagent coverage. This reagent coverage provides a rather weak link that is currently accepted, as the western blot is run under denaturing conditions and the ELISA is run under native conditions. Simple Western™ is a relatively new, automated, capillary western blot-based technology that allows for the separation, blotting, and detection of proteins. But, unlike traditional western blots, Simple Western™ is quantitative, allowing for the quantification of HCP content in biologic and vaccine samples. Antibody reagent validation is much more straightforward, as the reagent coverage can be directly linked between the 2D methodology and Simple Western™, as they are both run under denatured and reduced conditions. Methods: Herein we describe the development of a capillary western blot method to quantify the HCP content in samples generated using a Vero cell line for the production of an investigational live virus vaccine candidate (V590) for Coronavirus Disease-2019 (COVID-19). The HCP content in COVID-19 vaccine samples was evaluated using three methods: the new capillary western, the gold standard ELISA, and SDS-PAGE. Results/Conclusions: Strong agreement was observed in the HCP content data between the capillary western and SDS PAGE methods, whereas the ELISA HCP data were outliers, suggesting that the capillary western is generating HCP concentrations closer to the true concentration. This is the first report of using capillary western technology in analyzing HCP in vaccine samples.

## 1. Introduction

Host cell expression systems, such as Vero, *Saccharomyces cerevisiae*, and Chinese Hamster Ovary (CHO) cells, are used in biopharmaceutical production to generate significant masses of biological molecules, ranging from live viruses to recombinant proteins. Unfortunately, these host cell systems also produce endogenous host cell proteins (HCPs), including those critical for cell viability, protein expression, and metabolite usage. It has been demonstrated that HCP in biopharmaceutical drug products can pose potential safety risks to patients including, but not limited to, adjuvant effects, immunogenicity, and/or product stability [1,2,3]. Regulatory guidance has established that HCPs are considered process-related impurities and clearance levels should be set based on pre-clinical and clinical experience [4,5,6,7].

Regulatory guidance stresses that it is impossible to set acceptable HCP acceptance criteria limits that encompass all biotechnology products, as the HCP will vary qualitatively and quantitatively depending on the biological modality and the expression system [8]. For example, the AstraZeneca COVID-19 vaccine, ChAdOx1, was grown in Human embryonic kidney 293 cells (HEK-293), and an HCP limit was set at ~30,000 ng HCP/mg total protein [9], whereas therapeutic antibody production, generally grown in CHO, typically has HCP limits set between 1–100 ng HCP/mg total protein [10]. These differences are likely acceptable for a few reasons: purification capabilities, dosage differences, and administration frequency [11].

Despite the less stringent criteria for HCP in vaccine products, HCPs are still required to be extensively characterized due to their diverse nature and the inability to predict the effect any individual HCP would have on patient safety and product efficacy and stability [9,12]. Regulatory guidance has suggested two approaches to ensure HCP clearance: validation and routine monitoring [4,8]. In brief, a production process may be validated that demonstrates host cell protein levels are consistently cleared in the final product, or host cell protein content can be routinely monitored across batches to ensure clearance in the final product, respectively [4,8]. Alternatively, a combination of these approaches can also be used. Thus, methods must be established to quantify and characterize residual HCPs in vaccine drug products.

Two of the most used methods to analyze HCP are enzyme-linked immunosorbent (ELISA) and mass spectrometry (MS) [10,13]. In addition, manual western blotting and sodium dodecyl sulfate polyacrylamide gel electrophoresis (SDS-PAGE), including two-dimensional SDS PAGE and 2D-DIGE (different gel electrophoresis), are employed as qualitative tools to characterize HCP in vaccines [4,11,14]. However, there are many drawbacks associated with these techniques regarding HCP quantification and the practicality of their application. For example, MS is an ascending technique, but it is expensive and requires skilled operators, and manual western blotting and 2D DIGE are strictly qualitative techniques and are quite labor-intensive. Therefore, MS, manual western blotting, and 2D DIGE are generally only used for HCP extended characterization work when vaccine drug products are under investigation for atypical HCP content. In general, ELISA is the main technique used by the industry and is accepted by regulatory agencies to quantitate HCP for biopharmaceutical drug product release.

One of the drawbacks of using ELISA for HCP quantification is the requirement to qualify the anti-HCP antibody critical reagent. After the in vivo generation, anti-HCP polyclonal antibodies need to be validated for their wide coverage against HCP in the null lysate (i.e., lysate without the expression of the molecule of interest). This is typically addressed by performing 2D SDS PAGE where one gel is probed with an electrophoresis gel stain (i.e., Silver stain, SYPRO^TM^ Ruby, etc.) and the other gel is blotted with the anti-HCP polyclonal antibody [4,15]. Alternatively, 2D-DIGE can be used in place of the electrophoresis stained 2D gel [14,16]. The antibody coverage is calculated by qualitatively comparing the number of spots observed in the stained gel/2D-DIGE and western blot [4]. This reagent method validation is less than ideal, as 2D SDS PAGE and 2D western blots are run under denaturing and reducing conditions while ELISA is run under native conditions. Therefore, some antibodies may not recognize HCP epitopes in the native form and vice versa. Hence, it is desirable to have an alternative quantitative HCP assay that can be run under denaturing and reducing conditions to align with the anti-HCP antibody coverage qualification.

Western blot is an attractive method to perform this alternative HCP assay, as it can bridge the gap between the polyclonal antibody reagent qualification and use of antibodies in the ELISA method. However, as previously discussed, the current manual western blot is very time-consuming, labor-intensive, and not quantitative. Therefore, a faster, more automated, and quantitative western blot method is highly desirable.

Nguyen et al. have described an automated capillary electrophoresis (CE)-sized based western blot called Simple Western^TM^ (SW) [17]. We have evaluated this technology for qualitative and quantitative analyses of the Spike protein (S protein) in our V590 COVID-19 vaccine [18]. More recently, Pearson et al. published an initial evaluation using SW technology to quantitatively analyze HCP in CHO and Vero cell lines without sized-based separation [19]. Herein, we describe a more extensive development and understanding of an SW method for HCP quantitation in our V590 COVID-19 vaccine candidate produced in Vero cell line, and the results are compared with standard-method ELISA and SDS PAGE.

## 2. Materials and Methods

### 2.1. Commercial Reagents

Secondary antibody (anti-goat-horseradish peroxidase [HRP] conjugate), dithiothreitol (DTT), molecular weight fluorescent standards, luminol-S, hydrogen peroxide, 10× sample buffer, milk-free antibody diluent, wash buffer, 66–440 kDa plate pre-filled with stacking matrix and separation buffer, and capillaries were purchased from ProteinSimple^TM^ (Santa Clara, CA, USA). The primary antibody for the Vero cell line was affinity purified anti-Vero (Goat anti-Vero HCP 2G, Catalog# VC977-AF) purchased from Cygnus Technologies (Southport, NC, USA). The Vero HCP reference standard was produced in-house from the null Vero cell, and its concentration was measured by protein assay. Nunc™ MicroWell™ 96-Well Microplates, 3,3′,5,5′ tetramethylbenzidine (TMB) solution, Invitrogen NuPAGE SDS PAGE 4–20% bis-tris 1.0 mm gels, and Pierce™ 660 nm Protein Assay Reagent were purchased from Thermo Scientific (Waltham, MA, USA).

### 2.2. V590 COVID-19 Vaccine HCP Samples from Vero Cell

The V590 COVID-19 vaccine candidate was produced using a live-attenuated, replication-competent, recombinant vesicular stomatitis virus (rVSV) where the envelope VSV glycoprotein (G protein) gene was replaced with the S protein [20]. The downstream purification process for V590 production has been previously described [21]. In brief, the V590 COVID-19 vaccine was produced in Vero cells in a single-use bioreactor containing Cytodex-1 gamma irradiated microcarriers (Cytiva). At the end of fermentation growth, the cell culture harvested viral fluids (HVFs) were clarified using a 3 mm|0.8 mm filter. The clarified bulk material (CB) was purified using Capto^TM^ Core 700 resin chromatography (Cytiva). The Capto^TM^ Core flowthrough product pool (CCP) underwent an ultrafiltration/diafiltration (UF/DF) step where the material was concentrated, producing ultrafiltration concentrated retentate (UFCR), and then buffer exchanged, yielding ultrafiltration product (UFP). The permeate produced from the UF/DF step was referred to as ultrafiltration diafiltered permeate (UFDP). Recombinant human serum albumin was added as a stabilizer to the UFP, and it was sterile filtered in series through a 0.4 micron filter and a 0.2 micron filter to produce the formulated drug substance (DS). A flow chart has been provided for reference (Figure S1).

### 2.3. Detection and Quantitation of HCP Using SW in Live Virus COVID-19 Vaccine Samples

Vaccine samples were diluted in 1× sample buffer ranging from 5× to 25×. Internal Vero HCP reference standard was used and was serially diluted in sample buffer to construct calibration curve ranging from 3.20 µg/mL to 50.0 µg/mL. Then 2× master mix (without fluorescent markers) containing DTT and 1× sample buffer (ProteinSimple^TM^) was mixed in a 1:1 volume ratio with diluted vaccine samples. The samples were heated for 10 min at 90 °C. The samples were equilibrated to room temperature for 5 min, spun down for 2–3 s, and transferred into 66–440 kDa plates. All reference standards and system suitability samples (SSSs) were loaded onto the plate in duplicate. The primary antibody, affinity purified anti-Vero, was diluted 200× (5 µg/mL) in milk-free antibody diluent. The primary antibody incubation time was 45 min, and the secondary HRP incubation time was 30 min. The stacking gel was loaded into the capillaries for 12 s, and the sample injection time was 15s. Default vendor conditions were applied to other SW parameters. Data were analyzed using Compass for Simple Western^TM^ Version 6.2.0 software (ProteinSimple^TM^). All observed peaks within each electropherogram were integrated using baseline fit and dropline analysis.

### 2.4. Spike Protein SW Method

The spike protein (S protein) SW method was previously described in extensive detail [18]. Briefly, S protein reference standard material (ABclonal, Cat #RP01260MT) and vaccine samples were treated with 2× master mix solution prepared from the EZ Std kit Pack 1 12−230 kDa (ProteinSimple^TM^), which contains SDS and DTT. The treated samples were heated at 90 °C for 10 min to denature and reduce samples. Diluted primary anti-S-protein antibody (Sino Biological) was used for probing the S protein. All samples and reagents were loaded onto 12−230 kDa plates. The plates were analyzed using the SW instruments (i.e., Wes and Jess). All data were analyzed using the Compass for Simple Western^TM^ Version 6.2.0 software (ProteinSimple^TM^).

### 2.5. HCP ELISA

The Vero HCP reference standard (the same reference standard used in SW) range was from 5–2000 ng/mL diluted in assay buffer containing 11 mM Potassium Phosphate, 9% Sucrose, 1% BSA, and pH 7.5. The vaccine sample was diluted 1:200 and 1:400 in assay buffer. Samples, reference standard, and control were added into an ELISA plate coated with anti-Vero HCP antibody. Then, anti-Vero conjugated with HP was added for detection and incubated for 2 h at room temperature. After four washes, TMB solution was added into the plate and incubated for 30 min at room temperature. Finally, stop solution was added, and the plate was read using a Spectramax plate reader at 450 nm absorbance within 30 min. The data were analyzed using a quadratic fit with SoftMax^®^ Pro Version 7.1.2 or equivalent, and the zero standard was masked to remove background subtraction.

### 2.6. SDS PAGE

All samples were diluted in Laemmli reducing buffer containing DTT and heated at 70 °C for 10 min. The samples were loaded into an Invitrogen NuPAGE^TM^ SDS PAGE 4–20% bis-tris 1.0 mm gel system. The SDS PAGE gel separation was performed in Invitrogen 3-(N-morpholino)propanesulfonic acid (MOPS) running buffer at constant 125 V for 1 h and 40 min. The gels were fixed with 50% methanol/7% acetic acid for 2 h. After fixation, the gels were washed with water, and SYPRO^TM^ Ruby stain (ThermoFisher Scientific Waltham, MA, USA) was added and incubated overnight. Then the gels were destained with 10% methanol/7% acetic acid for 40 min and rinsed twice with water for 5 min each. The gels were scanned with a Bio-Rad Gel Doc^TM^ EZ Imager, and quantitation was performed using Bio-Rad Image lab^TM^ 5.2.1 software.

### 2.7. Total Protein Assay

Commercial protein assay Pierce™ 660 nm was used to measure the total protein concentration in the vaccine samples. Briefly, the assay was performed in 96-well plates where BSA reference standard was prepared from 50–400 μg/mL using 10 mM Tris-HCl, pH 7.2 as a diluent; 0.1 mg/mL BSA was used as a system suitability sample (SSS), and 20 mL of each diluted reference standard, SSS, and sample were transferred to a Nunc™ MicroWell™ 96-Well Microplate (Thermo Scientific) using a multichannel pipette in triplicate. Lastly, 200 mL of Pierce™ 660 nm Protein Assay Reagent (Thermo Scientific) was added to each well containing reference standard, SSS, and samples using a multichannel pipette. Each plate was incubated with a lid for 5 min at room temperature, and the absorbance was read at 660 nm using a SpectraMax M2^®^ plate reader (Molecular Devices, San Jose, CA, USA). The data were analyzed using SoftMax^®^ Pro software Version 7.1.2.

## 3. Results and Discussion

### 3.1. HCP Capillary Western Method Assay Development

**Figure 1:**
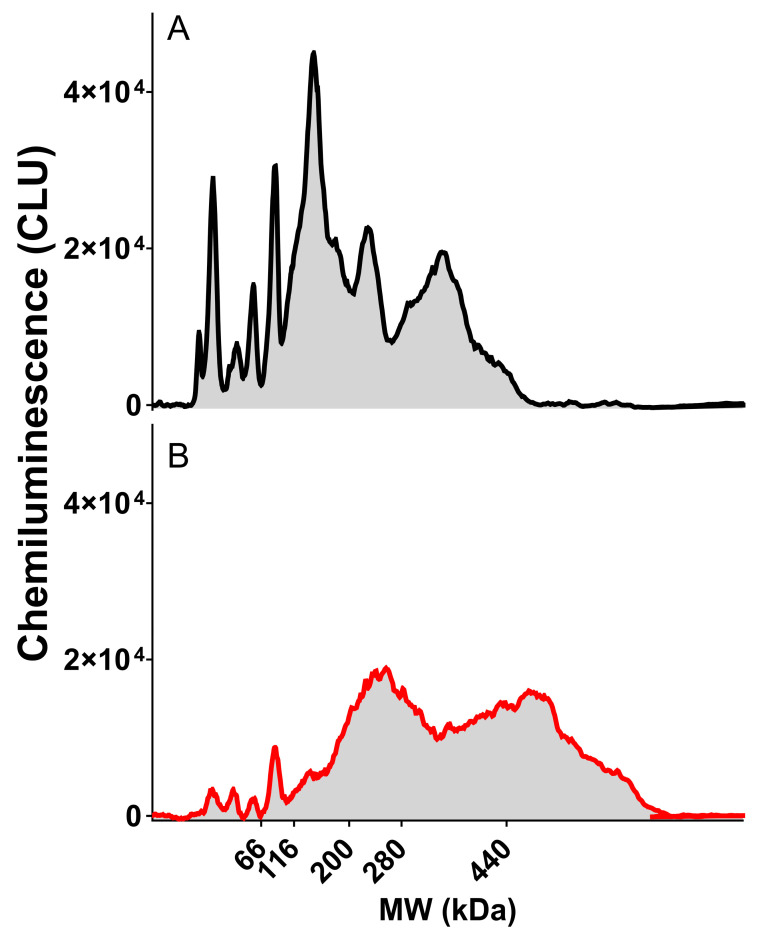
Example electropherogram traces and their peak area integration showing the broad MW range of HCP present in Vero cell in the (**A**) Vero HCP reference standard material at 25 μg/mL and (**B**) V590 COVID-19 UFP sample.

An anti-Vero cell primary antibody is critical for our immuno-based HCP SW assay. As there were time constraints in generating our own anti-Vero HCP polyclonal antibody, the commercial antibodies needed to be screened. Cygnus Technologies was the only commercial vendor that could be identified that produced an anti-Vero HCP antibody, so it was assessed against the internal HCP reference standard and UFP process intermediate sample (Figure 1). A broad molecular weight (MW) range of HCP was detected in both the reference standard and sample. Also, the peak profiles and response intensity were different between the reference standard and sample, as the identity and quantity of HCP in the reference standard and sample were not necessarily identical. Dropline integration was used for analysis, as shown in the shaded area under the curve (Figure 1). The sensitivity of the assay was enhanced by increasing the capillary injection time and by increasing the primary antibody incubation time (Figure S2). The specificity of the assay was improved by removing the fluorescent marker from the 2X master mix solution used for the final 1:1 dilution, as its components were cross-reactive to the anti-Vero HCP antibody (Figure S3).

**Figure 2:**
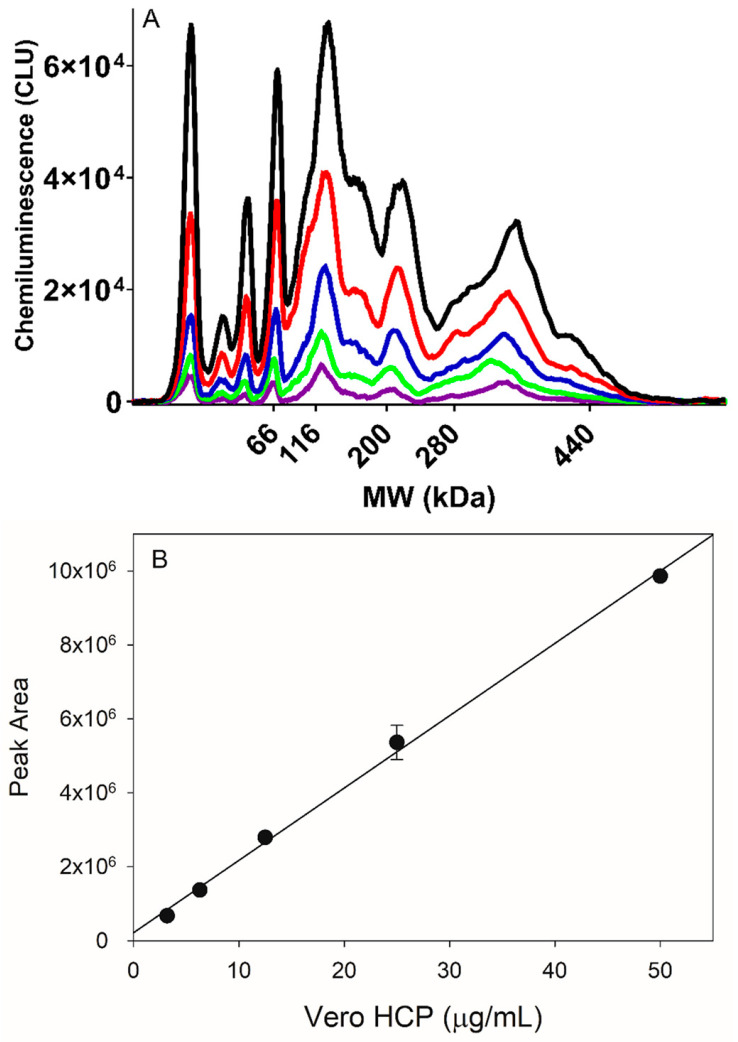
(**A**) Electropherograms of serial dilution of reference standards (purple: 3.20 μg/mL, Green: 6.25 μg/mL, Blue: 12.5 μg/mL, Red: 25.0 μg/mL and Black: 50.0 μg/mL); (**B**) the HCP standard curve from 3.20–50.0 μg/mL with r^2^ > 0.990.

For quantification, an internal null Vero cell reference standard was used to establish a linear standard curve range from 3.20–50.0 mg/mL with a coefficient of determination, R^2^ > 0.990 (Figure 2A,B). The precision and accuracy of the method were assessed to understand its performance. Precision was evaluated by running the same DS sample across multiple days, analysts, instruments, reagents, and capillaries, and it was determined to have a % RSD < 15% (n = 22). Accuracy was evaluated by spiking the internal null Vero cell reference standard into DS at four different spiking levels: 6.25 µg/mL, 12.5 µg/mL, 25.0 µg/mL, and 50.0 µg/mL. The average recoveries for each of the spike levels across four runs (n = 4 for each spike level) were within 100% ± 20%, which was acceptable for this assay. Based on the linearity, accuracy, and precision data, the estimated limit of quantitation (LOQ) and limit of detection (LOD) for this method are ~3.2 µg/mL and ~1.6 µg/mL, respectively.

Compared to ELISA, the sensitivity of the HCP SW assay is inferior by up to three logs (ng/mL vs. µg/mL), likely due to two reasons [22]. One, the HCP SW method is a sized-based separation where signal to noise (S/N) concerns could be observed for some of the lower abundant protein bands in the sub µg/mL range as the SW HCP signal is spread across a wide MW range. Whereas, in the ELISA, which has no sized-based separation, the signal is from the concentrated population of all HCP (e.g., similar to a dot blot, but in the native state), and greater S/N is observed in the sub µg/mL range, allowing for accurate quantification in that range. Two, the sample volume that can be loaded into the SW capillaries is limited compared to the ELISA; it is estimated that the SW sample injection volume is ~60 nL compared to ~100 µL on an ELISA plate. From a protein mass perspective, for a 100 ng/mL sample, this would translate to 10 ng protein mass in the ELISA versus 0.006 ng protein mass in the SW. This mass difference likely has a significant impact on the LOQ of the HCP SW method. Although the sensitivity of the HCP SW assay is inferior to the ELISA methodology, it is suitable for the HCP detection in V590 COVID-19 vaccine samples.

**Figure 3:**
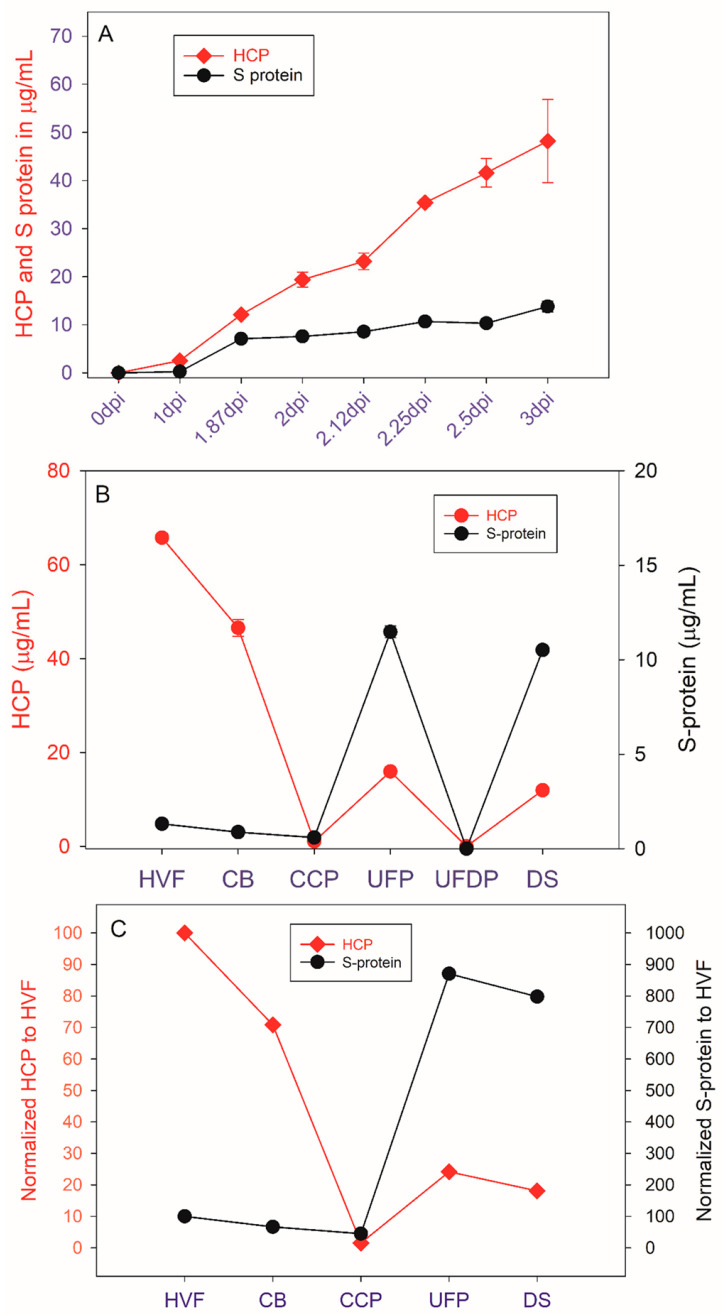
Comparison between HCP level and S protein production during (**A**) viral growth in bioreactor from 0 to 3 dpi; (**B**) HCP concentration level during V590 COVID-19 purification process; and (**C**) the percentage of HCP level clearance from HVF to DS as compared to the S protein production.

**Figure 4:**
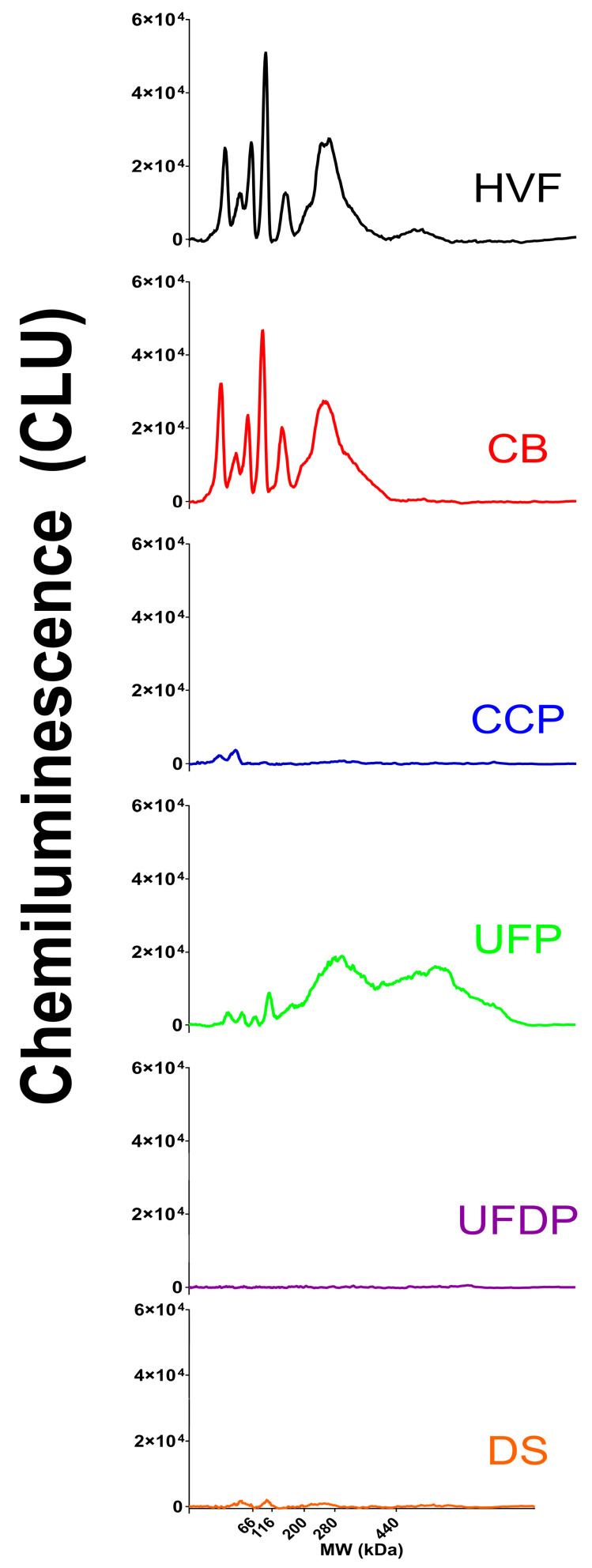
HCP electropherograms of each V590 COVID-19 purification step from HVF to DS showing the HCP MW distribution profiles across the purification process. The HCP electropherogram profiles during viral growth in bioreactor are similar to HVF.

HCP SW testing in COVID-19 vaccine production spike protein (S Protein) and HCP content were assessed throughout the COVID-19 vaccine production process from upstream Vero cell culture and viral infection through downstream viral purification to monitor viral particle production and purification (Figure 3). A previous study by Gillespie et al. demonstrated that the S protein concentration correlated well with the rVSV nucleoprotein (i.e., the desired rVSV particle) and potency; so, herein, it was used as a surrogate for rVSV purification along with the HCP clearance [18]. In addition, the MW distributions of the HCP profiles were examined at the various purification process steps to understand any differences in the HCP populations at each of the steps (Figure 4).

The rVSV viral seed was used to infect Vero cells, and the concentration of S protein (i.e., rVSV) and HCP were monitored for up to 3 days post infection (dpi) (Figure 3A). As expected, the concentration of rVSV and HCP increased with the infection incubation time. However, virus production had a rather insignificant increase after 2 dpi, whereas HCP continued to increase, quite significantly, past 2 dpi, suggesting there was an optimum harvest time to balance maximum virus production and minimum HCP levels.

In the downstream purification process, a large concentration of HCP with a wide MW range of HCP was observed in the HVF material (Figure 3B and Figure 4). After the initial HVF step, a ~30% reduction in similarly MW distributed HCP was observed after clarification, CB, with minimal loss of the rVSV, suggesting clarification assists with HCP removal while not impacting rVSV content (Figure 3B,C and Figure 4). The Capto^TM^ Core chromatography step, CCP, had high selectivity for HCP, but not for the rVSV, as it removed almost all of the HCP while not impacting the rVSV content (Figure 3B,C). This was likely because the small HCP entered the activated pores of the multimodal chromatography resin, while the larger VSV could not, allowing for the selective elution of the desired rVSV in the flowthrough material. Subsequently, the CCP material underwent UF/DF to diafilter and concentrate the material, resulting in an increase in the desired rVSV content and a slight increase in the residual HCP content at the UFP step (Figure 3B,C). In addition, the molecular weight distribution of the HCP in the ultrafiltration product had a slightly different profile than HVF and CB with a bias towards higher MW species (Figure 4). This observation is not surprising because the smaller HCP (<200 kDa) would have a greater tendency to enter the activated core of the Capto^TM^ Core chromatography resin step compared to the larger HCP. The permeate product, UFDP, contained very little HCP and rVSV (Figure 3B). Finally, a small reduction in HCP and rVSV content was observed in the final DS, as UFP was only diluted with rHSA (stabilizer) and sterile filtered to produce the DS (Figure 3B,C). The final purification process when normalized to the HVF step resulted in a 5-fold reduction in HCP concentration, while the rVSV increased ~80-fold, suggesting a very efficient purification process.

The HCP SW method, along with the S protein SW method, was very useful in upstream and downstream process optimization to identify conditions to reduce HCP content while increasing rVSV content in the DS.

### 3.2. Comparison of HCP SW and ELISA Testing

Since ELISA is the gold standard for HCP analysis, a comparison with the HCP SW assay was conducted. Several V590 UFP lots were run in both ELISA and HCP SW, and the results were compared (Table A1). Surprisingly, the ELISA HCP concentrations were ~5–15× lower than SW despite both methods using the same anti-HCP antibody and reference standard. The coverage of the Vero anti-HCP was verified to be >80% by 2D SDS PAGE (DIGE), eliminating potential antibody coverage issues. The SW has greater specificity compared to ELISA due to denaturing and reducing conditions minimizing matrix interference, so this could be a factor. However, the leading hypothesis for this discrepancy was the nature of the anti-HCP reactivity toward HCP in both assays, namely, native in ELISA (conformational epitopes) versus denatured and reduced in SW (linear epitopes) [10]. A third orthogonal HCP assay was required to further investigate this difference.

### 3.3. SDS PAGE HCP Assay

Since the five proteins that make up the V590 rVSV are known, nucleocapsid (N), Phosphoprotein (P), Matrix protein (M), RNA-dependent RNA polymerase (L), and Spike protein (S), a quantitative 1D SDS PAGE and a total protein assay were used as the third assay to further understand the ELISA and SW HCP discrepancy [23,24].

**Figure 5:**
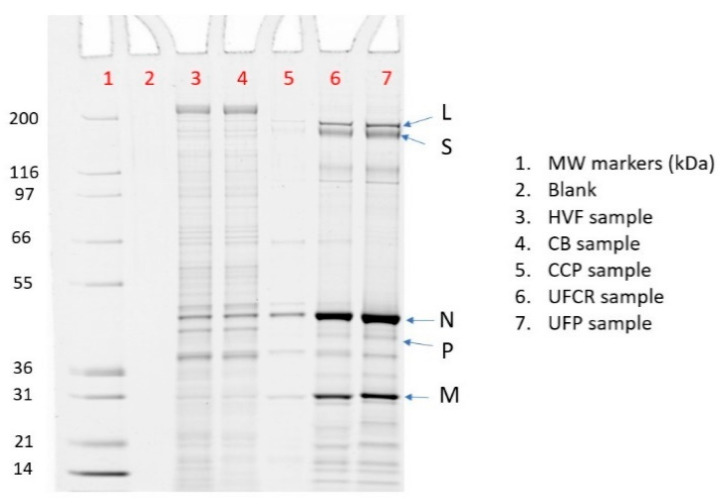
The SDS PAGE of V590 COVID-19 purification samples showing the rVSV purity increases from HVF to UFP (see Table 1). The DS was not included in % purity calculation since DS contains rHSA.

**Figure 6:**
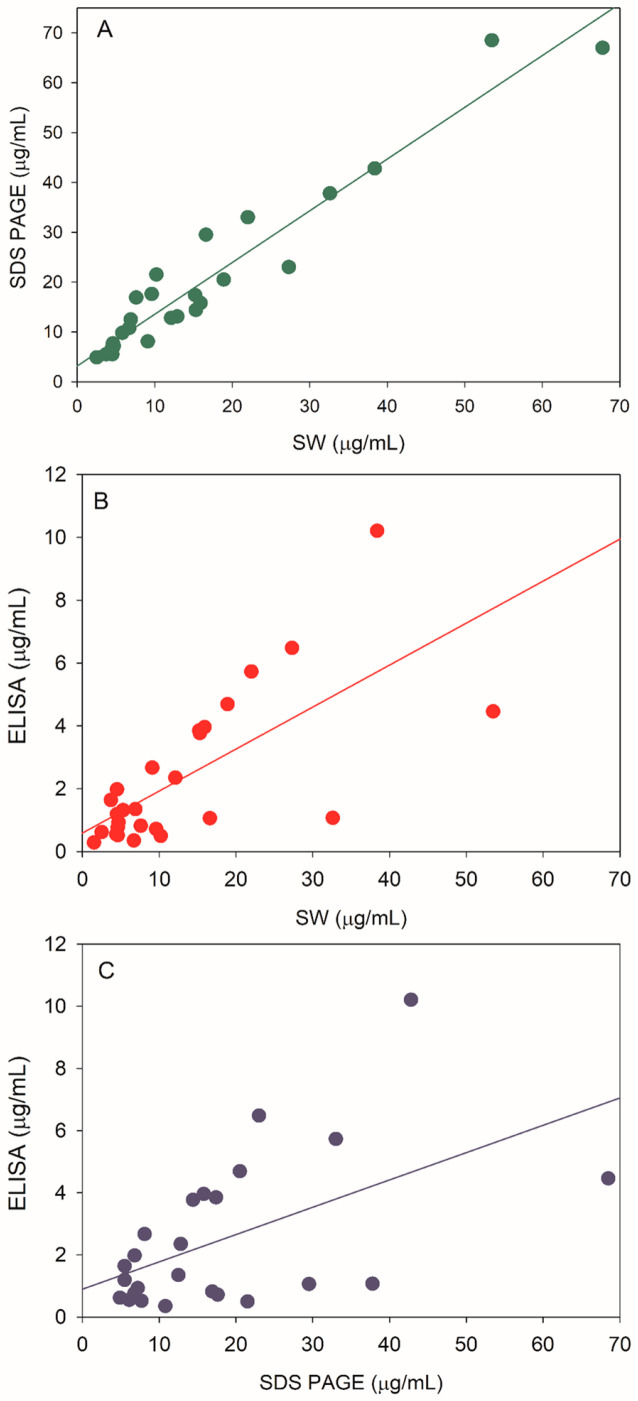
HCP correlations among three different assays: (**A**) SDS PAGE versus SW (R^2^ = 0.92); (**B**) ELISA versus SW (R^2^ = 0.60); and (**C**) SDS PAGE versus ELISA (R^2^ = 0.36).

The protein concentration of V590 process intermediate samples was measured via a Pierce^TM^ 660 nm protein content method (Table A1). The samples were normalized to the same protein concentration and loaded onto an SDS PAGE gel that was stained with SYPRO^TM^ Ruby stain (Figure 5). The five rVSV protein bands, N, P, M, L, and S, and the HCP bands were identified by in-gel digestion liquid chromatography-mass spectrometry/mass spectrometry (LC-MS/MS) (Figure S4). The % rVSV purity was quantitated by summing the peak areas of N, P, M, L, and S protein bands with respect to the total peak area of all bands (Figure S5). Unsurprisingly, in agreement with the HCP SW data (Figure 3), an increase in % purity was observed via SDS PAGE as sample intermediates proceeded through the downstream purification process (Table 1). The amount of HCP obtained from SDS PAGE was calculated by multiplying the % impurity (e.g. % Impurity= 100%—% purity) by the total protein concentration measured by a Pierce^TM^ 660 nm protein assay. HCP content was measured in twenty-nine different lots of UFP samples via SW, SDS PAGE, and ELISA to understand the correlation of the three different assays (Table A1, Figure 6). The data clearly demonstrate the best correlation was between SW and SDS PAGE (R^2^ = 0.92) compared to both SW versus ELISA (R^2^ = 0.60) and SDS PAGE versus ELISA (R^2^ = 0.36). Therefore, the HCP concentrations measured by the SW method are likely closer to the true HCP concentrations compared to ELISA.

**Figure 7:**
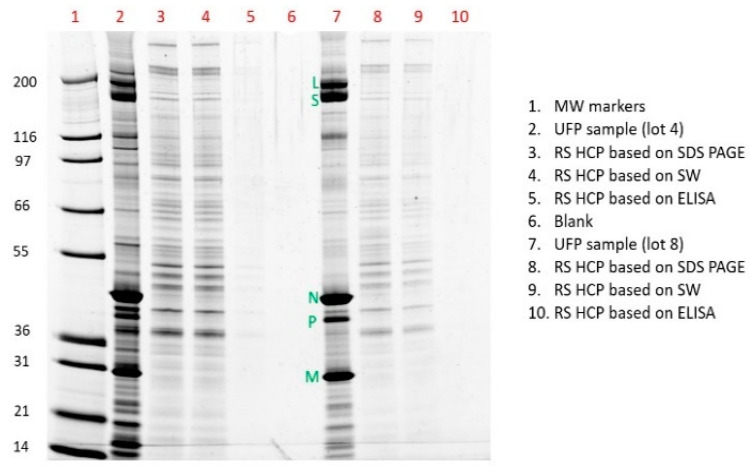
SDS PAGE profile for visual comparison between UFP sample and HCP reference standard (RS), loaded according to the measured HCP concentrations by SDS PAGE, SW, and ELISA for two different samples: lot 4 and lot 8 (from Table A1).

Lastly, the HCP concentrations measured by each assay, SW, ELISA, and SDS PAGE, from two different UFP lots were loaded onto a SDS PAGE gel and were qualitatively compared (Figure 7). A visual comparison of the signal intensities clearly demonstrates that the HCP level measured by SW and SDS PAGE are in agreement, suggesting they are likely closer to the true HCP numbers in the UFP lots than ELISA.

As hypothesized, this difference between SW and ELISA HCP concentration measurements is likely caused by the difference in anti-HCP antibody epitope recognition differences: linear versus conformational epitopes, respectively. The anti-HCP antibody reagent coverage validation is much more in alignment with SW than ELISA due to the denaturing conditions used in the reagent validation. Therefore, this entire data package, along with the stronger antibody reagent coverage validation link, suggests that SW is a much more appropriate method for measuring HCP in the V590 COVID-19 vaccine samples than ELISA. Furthermore, SW is simple and automated and has a fast data turnaround time (~3 h), allowing it to be used as a potential at-line process analytical tool (PAT).

## 4. Conclusions

Herein we have described using automated capillary western technology (Simple Western^TM^) to detect and quantitate HCP in COVID-19 vaccine production from upstream rVSV growth in Vero cells to its downstream purification. Pre-validation parameters, including linearity, accuracy, and precision, were evaluated, demonstrating suitable performance. The HCP SW method was compared with ELISA, and the observed concentration difference between the two methods was further investigated by measuring HCP content using SDS PAGE and a Pierce^TM^ 660 nm protein assay. The results demonstrated that HCP concentrations measured by SW were closer to SDS PAGE than ELISA, suggesting that SW is generating HCP concentrations closer to the true concentration. This is the first report of using SW technology in analyzing HCP in vaccine samples. The ability of SW to analyze HCP in viral vaccine process development could potentially offer an alternative, or perhaps a better choice, to the current industry-standard ELISA method. Besides the automation and ease of use, there are other potential advantages of HCP analysis using capillary western as compared to ELISA, including the direct link to the antibody reagent validation coverage and the greater specificity. One of the most important advantages is the stronger correlation of the critical anti-HCP antibody reagent validation coverage using 2D SDS PAGE gel and 2D manual western methods with capillary western compared to ELISA. Another advantage is the greater specificity of the capillary western in HCP analysis of unpurified samples compared to ELISA. The denaturing and reducing conditions help to minimize any matrix interference. There is only one disadvantage of the current SW technology compared to ELISA, and that is the sensitivity difference (µg/mL vs. ng/mL). Although the current sensitivity of the SW method is more than sufficient for HCP analysis in most vaccine products, it may not be low enough for DS products with high concentrations such as mAb therapeutic proteins. A lower LOQ for the capillary western method may still be possible if the HCPs are concentrated into one band, similar to a dot blot, by utilizing advanced stacking methods; however, additional method development would be required.

## Figures and Tables

**Table 1:** The % purity calculated via SDS PAGE of rVSV intermediate samples from Figure 5.

Lane	Sample	% rVSV	% L	% S	% N	% P	% M
3	HVF	16%	1%	2%	12%	0%	2%
4	CB	18%	1%	2%	14%	0%	2%
5	CCP	67%	5%	0%	50%	0%	11%
6	UFCR	78%	5%	8%	46%	2%	16%
7	UFP	80%	7%	8%	46%	2%	17%

## Data Availability

Data will be made available upon reasonable request through the corresponding author.

## Supplementary Materials

The following supporting information can be downloaded at https://www.mdpi.com/article/10.3390/vaccines12121373/s1: Figure S1: V590 downstream process flow chart; Figure S2: sensitivity enhancement; Figure S3: master mix fluorescent marker interference; Figure S4: SDS-PAGE band identification via in-gel digestion MS; and Figure S5: % rVSV purity calculation.

## Appendix A

**Table A1:** Comparison of HCP concentrations measured by SDS PAGE, SW, and ELISA (NT: Not tested).

UFP Sample	% rVSV Purity	Total Protein in µg/mL	rVSV in µg/mL	HCP (SDS PAGE) in µg/mL	HCP (SW) in µg/mL	HCP (ELISA) in µg/mL
Lot 1	40.3	22.0	8.90	13.1	12.9	NT
Lot 2	25.6	90.0	23.0	67.0	67.8	NT
Lot 3	47.5	72.0	34.2	37.8	32.6	1.07
Lot 4	60.2	172	104	68.5	53.5	4.46
Lot 5	74.8	70.0	52.4	17.6	9.60	0.720
Lot 6	17.5	40.0	7.00	33.0	22.0	5.73
Lot 7	53.1	49.0	26.0	23.0	27.3	6.48
Lot 8	77.5	131	102	29.5	16.6	1.06
Lot 9	77.2	24.0	18.5	5.50	3.70	1.64
Lot10	74.8	27.0	20.2	6.80	4.50	1.98
Lot 11	66.2	38.0	25.2	12.8	12.1	2.35
Lot 12	77.6	96.0	74.5	21.5	10.2	0.500
Lot 13	74.0	31.0	22.9	8.10	9.10	2.67
Lot 14	78.0	77.0	60.1	16.9	7.60	0.820
Lot 15	77.6	32.0	24.8	7.20	4.70	0.930
Lot 16	78.0	79.0	61.6	17.4	15.2	3.85
Lot 17	80.0	79.0	63.2	15.8	15.9	3.96
Lot 18	NT	48.5	NT	NT	1.50	0.290
Lot 19	78.4	50.0	39.2	10.8	6.70	0.350
Lot 20	81.9	69.3	56.8	12.5	6.90	1.35
Lot 21	80.8	75.0	60.6	14.4	15.3	3.77
Lot 22	62.5	114	71.3	42.8	38.4	10.2
Lot 23	90.2	69.0	62.2	6.80	4.60	0.770
Lot 24	92.0	69.0	63.5	5.50	4.50	1.19
Lot 25	82.9	120.	99.5	20.5	18.9	4.69
Lot 26	90.6	52.0	47.1	4.90	2.50	0.620
Lot 27	90.6	65.0	58.9	6.10	4.40	0.550
Lot 28	88.3	66.0	58.3	7.70	4.60	0.520
Lot 29	75.6	40.0	30.20	9.80	5.80	NT
