# Supplementary material for: Automated, Quantitative Capillary Western Blots to Analyze Host Cell Proteins in COVID-19 Vaccine Produced in Vero Cell Line
Source: Vaccines (Basel). 2024 Dec 5;12(12):1373. doi: 10.3390/vaccines12121373 (PMC11680091; doi:10.3390/vaccines12121373)
Supplement: Supplementary file 1 [file vaccines-12-01373-s001.zip › vaccines-3310871-supplementary.pdf]

Supporting Information for:

Automated, Quantitative Capillary Western Blots to Analyze Host Cell Protein Content in COVID-19 Vaccine Produced in Vero Cell Line

Paul F. Gillespie[1], Yanjie Wang[1], Kuo Yin[1], Emily Groegler[1], Nicholas Cunningham[1], Alyssa Q. Stiving[1], Jessica Raffaele[1], Natalia Marusa[1], Christopher M. Tubbs[1], John W. Loughney[1], Michael A. Winters[2], Richard R. Rustandi[1]*

1 Merck & Co., Inc., Rahway, NJ, USA Analytical Research Development
2 Merck & Co., Inc., Rahway, NJ, USA Vaccine Process Research & Development
* Correspondence: Paul.Gillespie1@merck.com; Tel.: (215) 993 2261 770 Sumneytown Pike, P. O. Box 4, WP42A-20 West Point, PA 19486, USA

Table of Contents: Page Number

I. V590 Downstream Process Flow Chart S2
II. Sensitivity Enhancement S2
III. Master Mix Fluorescent Marker Interference S3
IV. SDS-PAGE Band Identification via In-Gel Digestion MS S4
V. % rVSV Purity Calculation S5

I. COVID-19 Vaccine Downstream Process Flow Chart

The COVID-19 vaccine downstream process was discussed in detail in the Materials and Methods. A flow chart of the COVID-19 vaccine downstream process has been provided for reference (Figure S1).

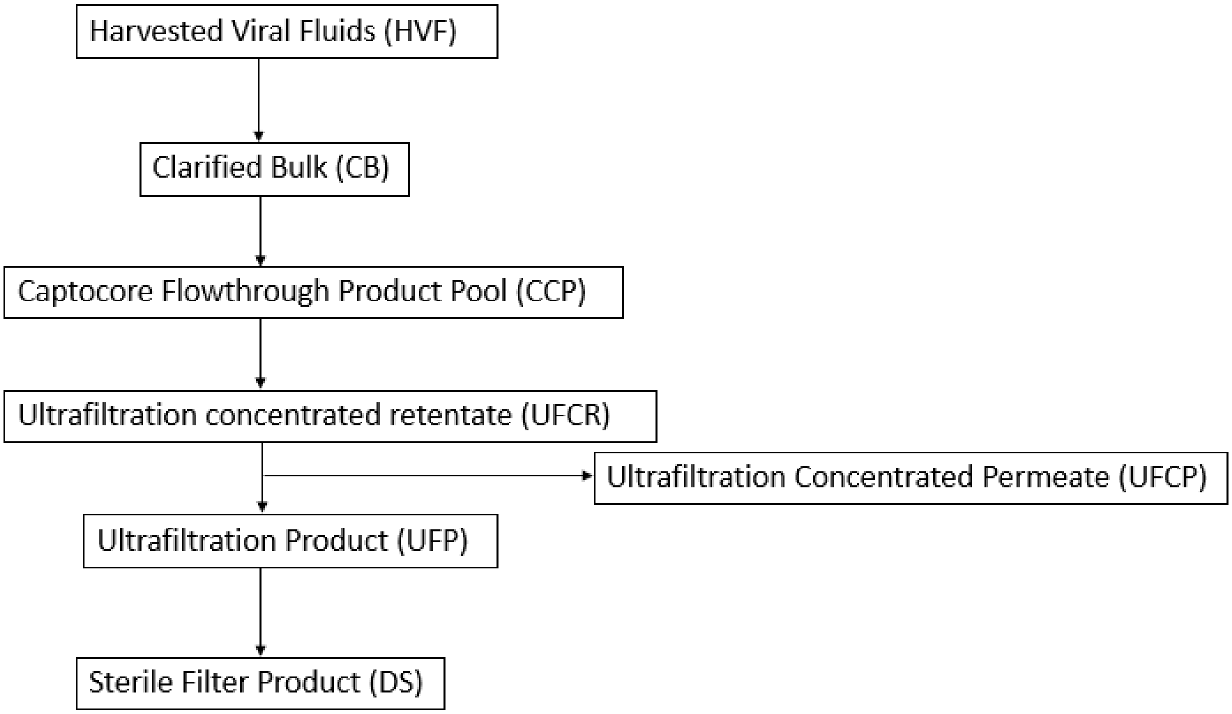

Figure S1: Flow chart of the COVID-19 vaccine downstream purification process.

II. Sensitivity Enhancement

50µg/mL Vero cell reference standard was analyzed with different capillary injection times (i.e. the amount of sample injected) and primary antibody incubation times to improve the sensitivity of the HCP SW assay. The sensitivity of the HCP SW assay was enhanced by increasing the capillary injection time from 8 sec to 15 sec and the primary antibody incubation time from 30 min to 45 min (Figure S2).

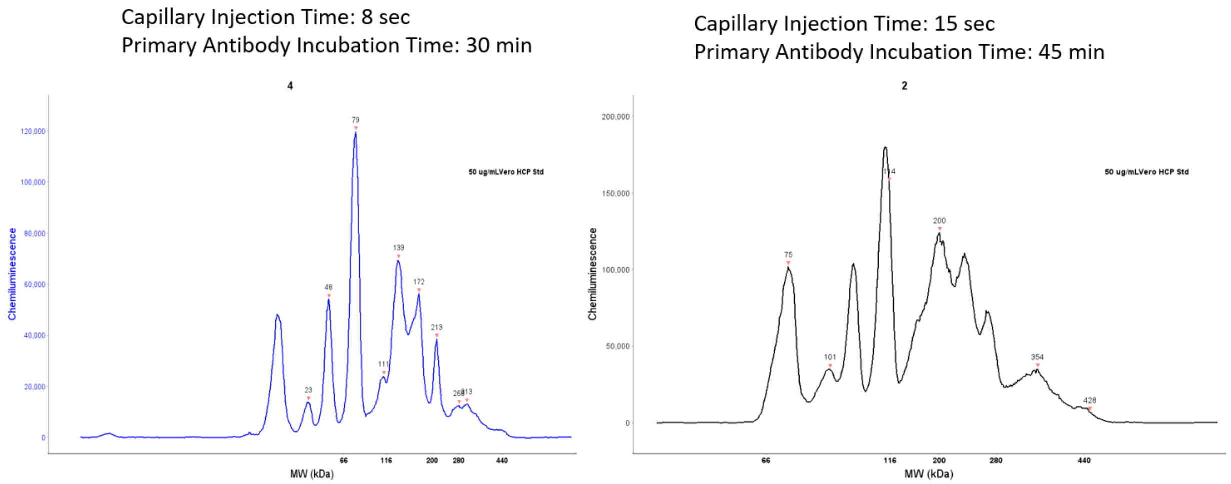

Figure S2: Electropherograms of 50ug/mL HCP standard with default capillary injection time and primary antibody incubation time (left) and increased capillary injection time and primary antibody incubation time (right).

III. <u>Master Mix Fluorescent Marker Interference</u>

Capto™ Core flowthrough product pool (CCP) sample was analyzed with and without the fluorescent marker from the 2X master mix solution and it was determined that the anti-Vero HCP antibody was cross reactive to it (figure S3). Therefore, the master mix fluorescent marker was removed from the final 2X master mix solution to improve the specificity of the HCP SW assay.

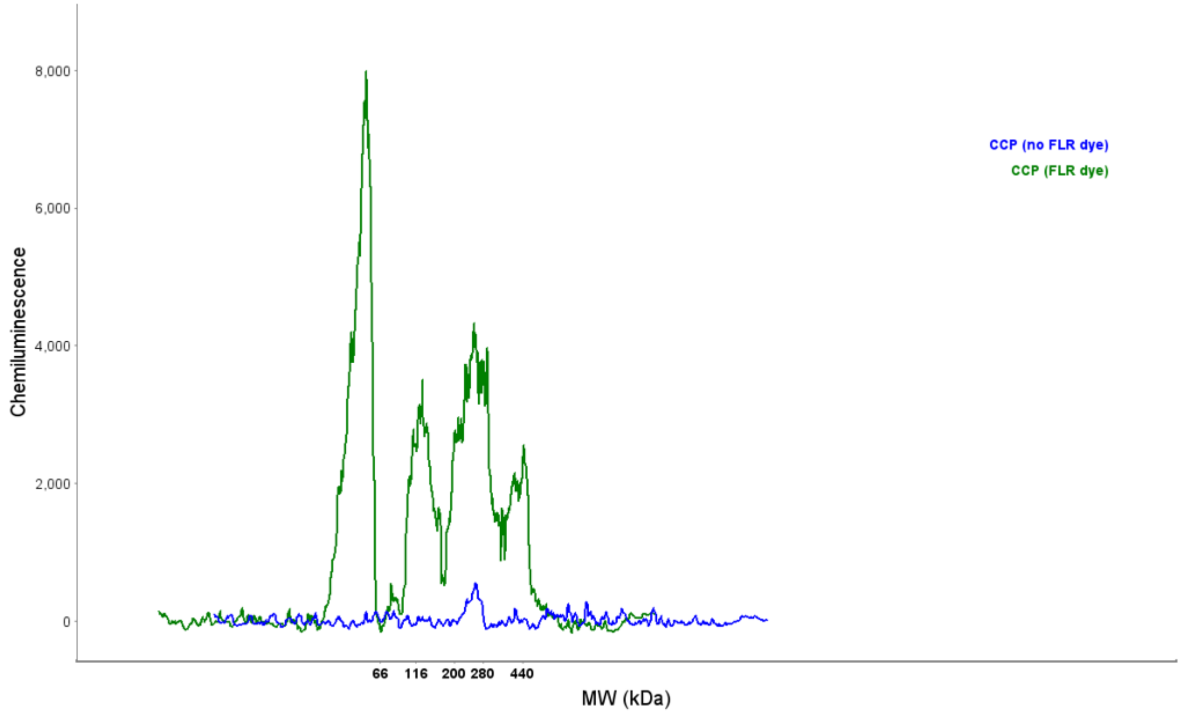

Figure S3: Electropherogram of Capto^{TM} Core flowthrough product pool (CCP) with no master mix fluorescent dye (no FLR dye) and with master mix fluorescent dye (FLR dye).

IV. SDS-PAGE Band Identification via In-Gel Digestion LC-MS/MS

For the SDS-PAGE HCP assay the five rVSV protein bands, N, P, M, L and S, and the HCP bands needed to be identified to ensure an accurate % purity calculation. UFP samples were run on an SDS-PAGE gel and stained with coomassie blue. Bands were excised and destained in an ammonium bicarbonate in acetonitrile solution. Then, the bands underwent reduction, alkylation and digestion with trypsin and analyzed via LC-MS/MS analysis using a thermo orbitrap exploris 480. Following LC-MS/MS analysis, the MS raw data files were analyzed using Mascot. The major rVSV proteins and HCPs were identified and utilized in the SDS-PAGE HCP assay (figure S4).

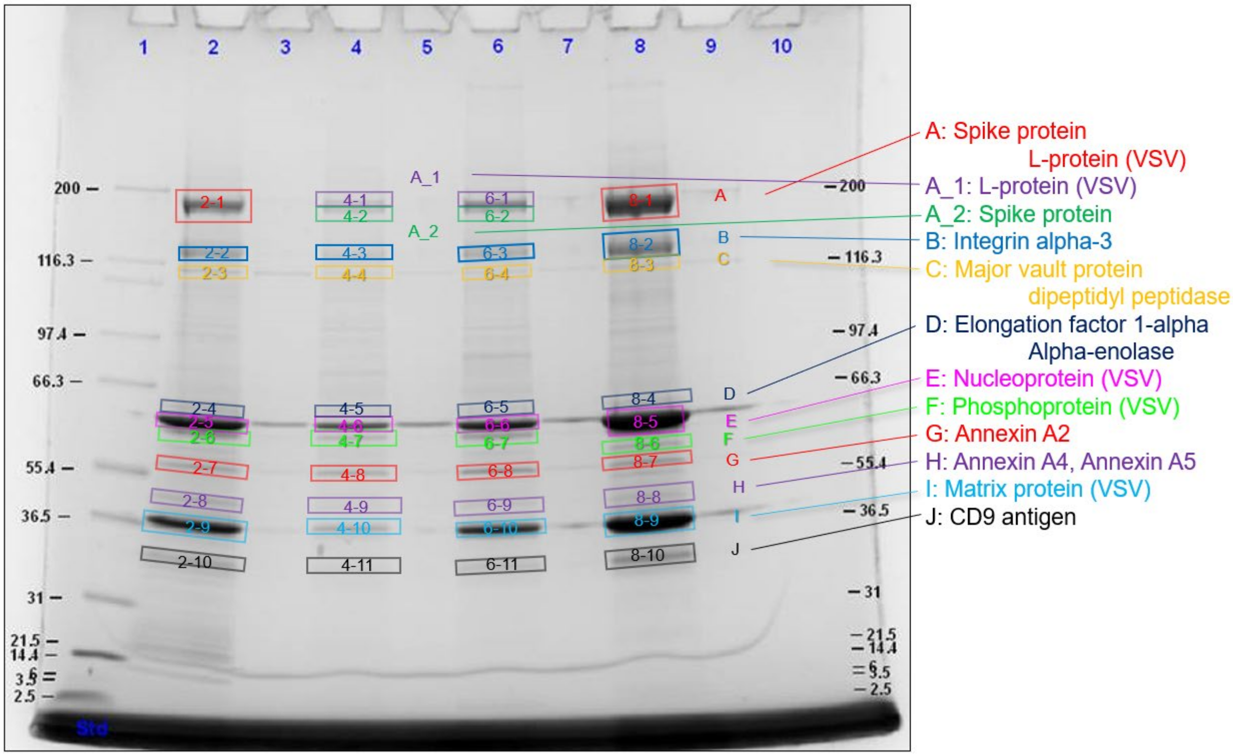

Figure S4: SDS-PAGE band identification, via LC-MS/MS, of the major rVSV proteins and HCPs in a UFP sample.

V. <u>% rVSV Purity Calculation</u>

The % rVSV purity was calculated by adding together the peak areas of each of the five rVSV protein bands, N, P, M, L and S, and dividing it by the total peak area of all bands (figure S5).

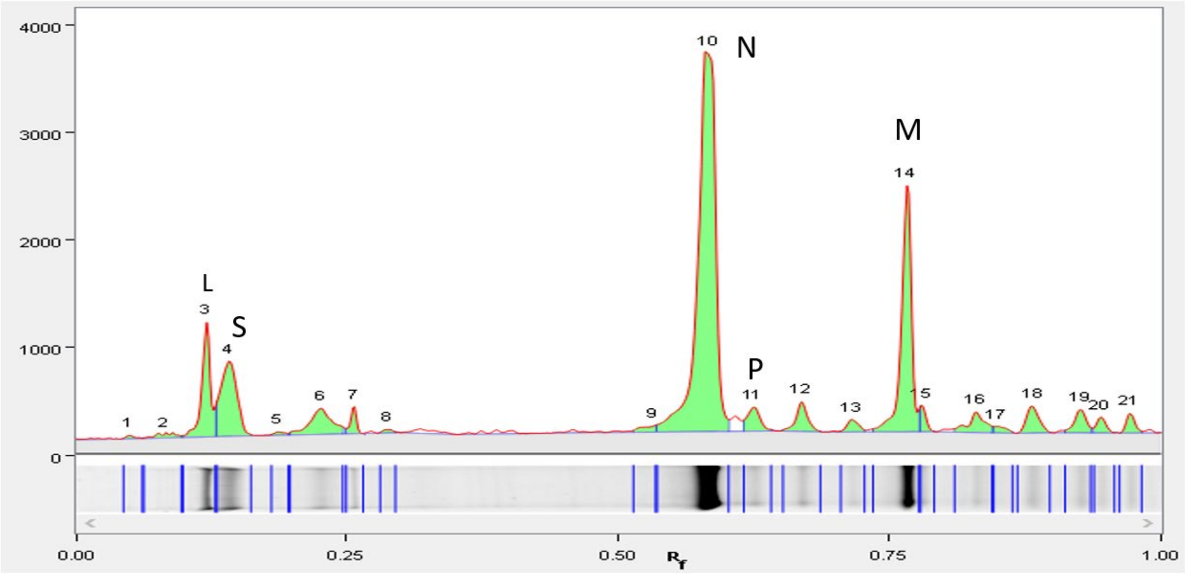

Figure S5: A band densitometry trace of a V590 UFP sample.
